# Supplementary material for: μLED‐based optical cochlear implants for spectrally selective activation of the auditory nerve
Source: EMBO Mol Med. 2020 Jun 29;12(8):e12387. doi: 10.15252/emmm.202012387 (PMC7411546; doi:10.15252/emmm.202012387)
Supplement: Supplementary file 1 — Appendix [file EMMM-12-e12387-s001.pdf]

Appendix

µLED-based optical cochlear implants for precise control of the auditory system

Alexander Dieter[1,2*], Eric Klein[3*], Daniel Keppeler[1], Lukasz Jablonski[1,4], Tamas Harczos[1,4], Gerhard Hoch[1,4], Vladan Rankovic[1,4,5], Oliver Paul[3,7], Marcus Jeschke[1,4,6], Patrick Ruther[3,7], Tobias Moser[1,2,4,8]

[1]Institute for Auditory Neuroscience and InnerEarLab, University Medical Center Göttingen, 37075 Göttingen, Germany

[2]Göttingen Graduate School for Neurosciences and Molecular Biosciences, University of Göttingen, 37075 Göttingen, Germany

[3]Department of Microsystems Engineering (IMTEK), University of Freiburg, 79110 Freiburg, Germany

[4]Auditory Neuroscience and Optogenetics Laboratory, German Primate Center, 37077 Göttingen

[5]Restorative Cochlear Genomics Group, Auditory Neuroscience and Optogenetics Laboratory, German Primate Center, 37077 Göttingen

[6]Cognitive Hearing in Primates Group, Auditory Neuroscience and Optogenetics Laboratory, German Primate Center, 37077 Göttingen, Germany

[7]BrainLinks-BrainTools, Cluster of Excellence, University of Freiburg, 79110 Freiburg, Germany

[8]Auditory Neuroscience Group, Max Planck Institute for Experimental Medicine, 37075 Göttingen, Germany

[9]Cluster of Excellence "Multiscale Bioimaging: from Molecular Machines to Networks of Excitable Cells" (MBExC), University of Goettingen, Germany

Appendix Table of Content:

Appendix Figure S1: Optical power of µLED-based oCIs
Appendix Figure S2: Response window of oCI evoked neural responses
Appendix Figure S3: Spatial spread of excitation
Appendix Figure S4: Tomogram of the oCI-implanted cochlea
Appendix Table S1: Statistics

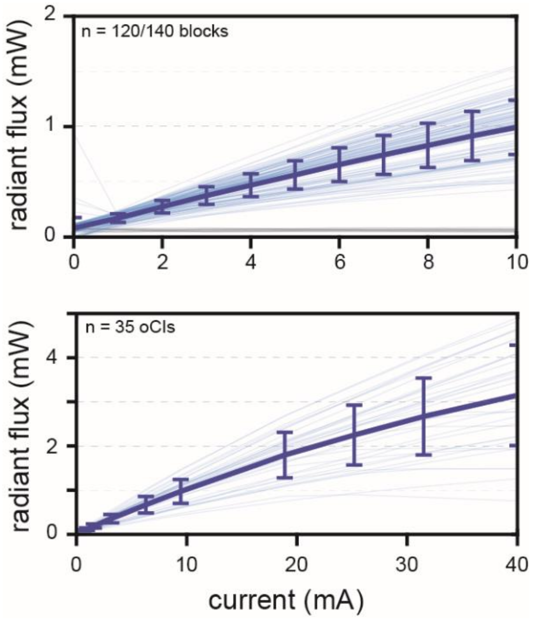

Appendix Figure S1: Radiant flux of µLED based oCIs. The radiant flux of four neighboring µLEDs (top) was estimated from the power of individual µLEDs measured before each experiment (up to 2.5 mA × 4; Fig. 1H), since the driving current of 10 mA was distributed over all four µLEDs. Radiant flux for all µLEDs on an oCI (bottom) was measured before each experiment. Solid lines indicate the mean, error bars the standard deviation of the mean. Non-functional µLEDs (which did not emit light) have been excluded from the estimation of blockwise radiant flux.

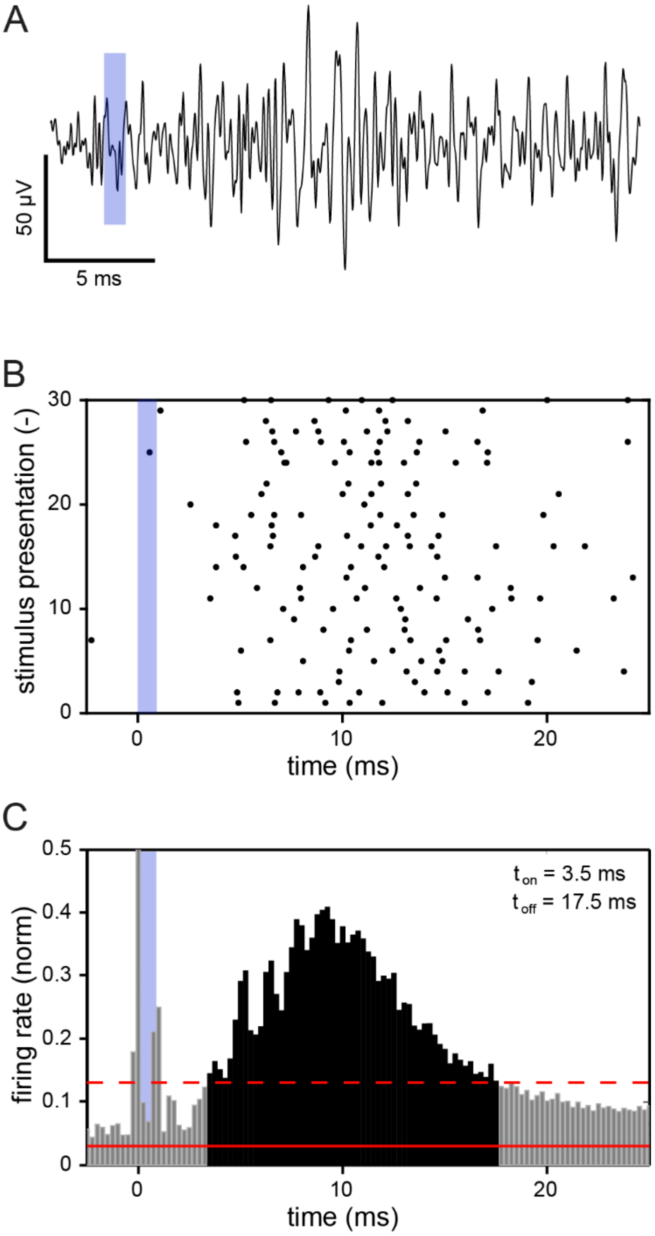

Appendix Figure S2: Response window of oCI evoked responses. (A) Filtered (0.6-6 kHz) trace of a multi-unit recorded from a single electrode in response to a 1 ms light pulse of ~ 3 mW. **(B)** Scatter plot of a multi-unit in response to a 1 ms light pulse of ~ 3 mW. Different trials (n = 30) are shown on the ordinate. **(C)** Peri-stimulus time histogram in response to SGN stimulation with 16 active µLEDs of an oCI at maximum radiant flux (~2.9 mW), composed of multi-units recorded from all recording sites. Stimulus presentation is indicated in blue. Solid and dashed red lines indicate the mean firing rate plus 3 standard deviations, respectively. Bin size was set to 0.25 ms, and the detected neural response is indicated in black.

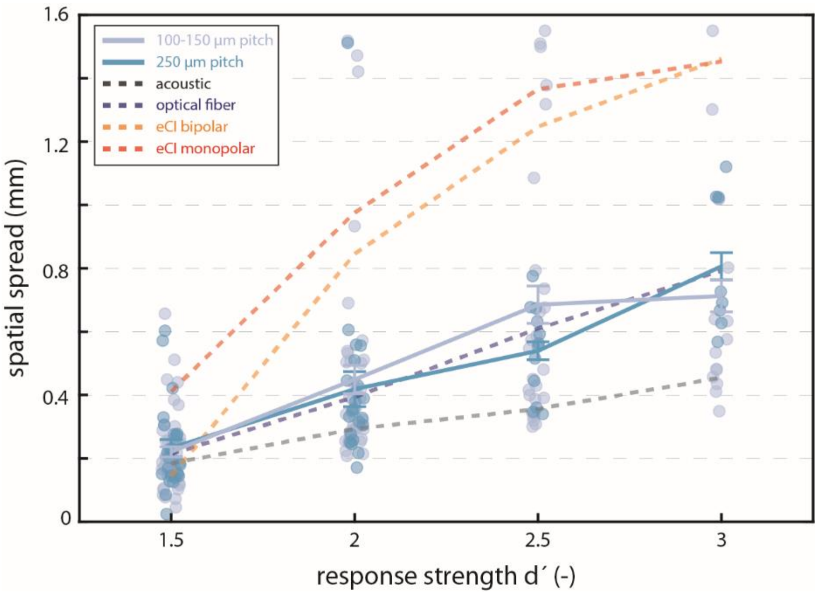

Appendix Figure S3: Spatial spread of excitation. Spread of ICC activity upon SGN stimulation with µLED-based oCIs (solid lines; mean ± standard error of mean), acoustic stimulation, laser-coupled optical fibers, as well as mono- and bipolar electrical stimulation with a clinical-style eCI (dashed lines). Data of acoustic stimulation and stimulation via optical fibers and electrical CIs is reprinted from Dieter et al (Dieter *et al*, 2019).

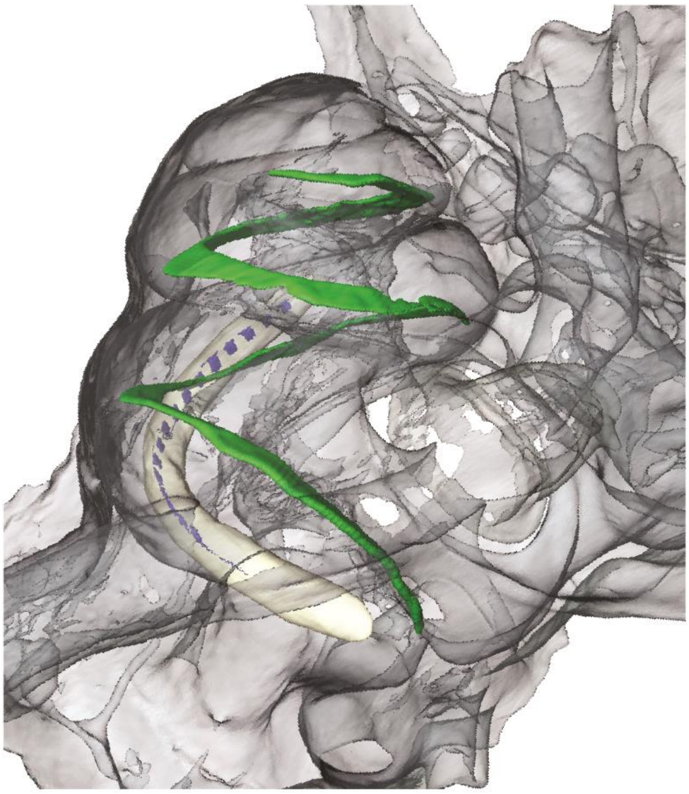

Appendix Figure S4: Tomogram of an oCI-implanted cochlea. X-ray based 3D reconstruction of a gerbil cochlea implanted with a 16-channel µLED-based oCI. Blue: µLEDs; yellow: Polymer carrier substrate and silicone encapsulation of oCI; green: basilar membrane. µLEDs have a size of 60×60 µm

Appendix Table S1: Statistical tests used and obtained significance of data presented in this manuscript.

Experiment	figure	statistical test	compared values	p-value
tonotopy (acoustic stimulation)	Fig. EV3	Pearson's correlation coefficient	recording depths vs. characteristic freuqencies	2.2x10^-77
response strength individual µLEDs	Fig. 2I	Wilcoxon Rank Sum	hearing vs. deaf gerbils	0.26
response strength blockwise µLEDs	Fig. 2I	Wilcoxon Rank Sum	hearing vs. deaf gerbils	0.80
response strength all µLEDs	Fig. 2I	Wilcoxon Rank Sum	hearing vs. deaf gerbils	0.43
response strength fiber stimulation	Fig. 2I	Wilcoxon Rank Sum	hearing vs. deaf gerbils	1
response strength of different emitters	Fig. 2I	ANOVA & post-hoc mult. comparison	individual µLED vs. blockwise µLED	9.9*10^-9
response strength of different emitters	Fig. 2I	ANOVA & post-hoc mult. comparison	blockwise µLED vs. all µLEDs	0.0096
response strength of different emitters	Fig. 2I	ANOVA & post-hoc mult. comparison	all µLEDs vs. optical fiber	0.0016
response strength of different emitters	Fig. 2I	ANOVA & post-hoc mult. comparison	all µLEDs *CatCh*-injected vs. all µLEDs WT	9.9*10^-9
number of active electrodes individual µLEDs	Fig. 2J	Wilcoxon Rank Sum	hearing vs. deaf gerbils	0.01
number of active electrodes blockwise µLEDs	Fig. 2J	Wilcoxon Rank Sum	hearing vs. deaf gerbils	0.11
number of active electrodes all µLEDs	Fig. 2J	Wilcoxon Rank Sum	hearing vs. deaf gerbils	0.14
number of active electrodes fiber stimulation	Fig. 2J	Wilcoxon Rank Sum	hearing vs. deaf gerbils	1
number of active electrodes of different emitters	Fig. 2J	ANOVA & post-hoc mult. comparison	individual µLED vs. blockwise µLED	6.8*10^-5
number of active electrodes of different emitters	Fig. 2J	ANOVA & post-hoc mult. comparison	blockwise µLED vs. all µLEDs	0.006
number of active electrodes of different emitters	Fig. 2J	ANOVA & post-hoc mult. comparison	all µLEDs vs. optical fiber	0.25
number of active electrodes of different emitters	Fig. 2J	ANOVA & post-hoc mult. comparison	all µLEDs *CatCh*-injected vs. all µLEDs WT	9.9*10^-9
threshold of neural activation	Fig. 2K	Wilcoxon Rank Sum	oCI (all µLEDs) vs. optical fiber	0.012
tonotopy (oCI activation - individual µLEDs)	Fig. 3B	Pearson's correlation coefficient	relative emitter location vs. relative best electrode	0.0007
tonotopy (oCI activation - blockwise)	Fig. 3B	Pearson's correlation coefficient	relative emitter location vs. relative best electrode	0.0000002
spectral spread - blockwise oCI activation	Fig. 3C	ANOVA & post-hoc mult. comparison	100-150 µm pitch vs. 250 µm pitch, d' = 1.5	0.81
spectral spread - blockwise oCI activation	Fig. 3C	ANOVA & post-hoc mult. comparison	100-150 µm pitch vs. 250 µm pitch, d' = 2.0	0.60
spectral spread - blockwise oCI activation	Fig. 3C	ANOVA & post-hoc mult. comparison	100-150 µm pitch vs. 250 µm pitch, d' = 2.5	0.22
spectral spread - blockwise oCI activation	Fig. 3C	ANOVA & post-hoc mult. comparison	100-150 µm pitch vs. 250 µm pitch, d' = 3.0	0.69
