# Supplementary material for: μLED‐based optical cochlear implants for spectrally selective activation of the auditory nerve
Source: EMBO Mol Med. 2020 Jun 29;12(8):e12387. doi: 10.15252/emmm.202012387 (PMC7411546; doi:10.15252/emmm.202012387)
Supplement: Supplementary file 2 — Expanded View Figures PDF [file EMMM-12-e12387-s002.pdf]

Expanded View Figures

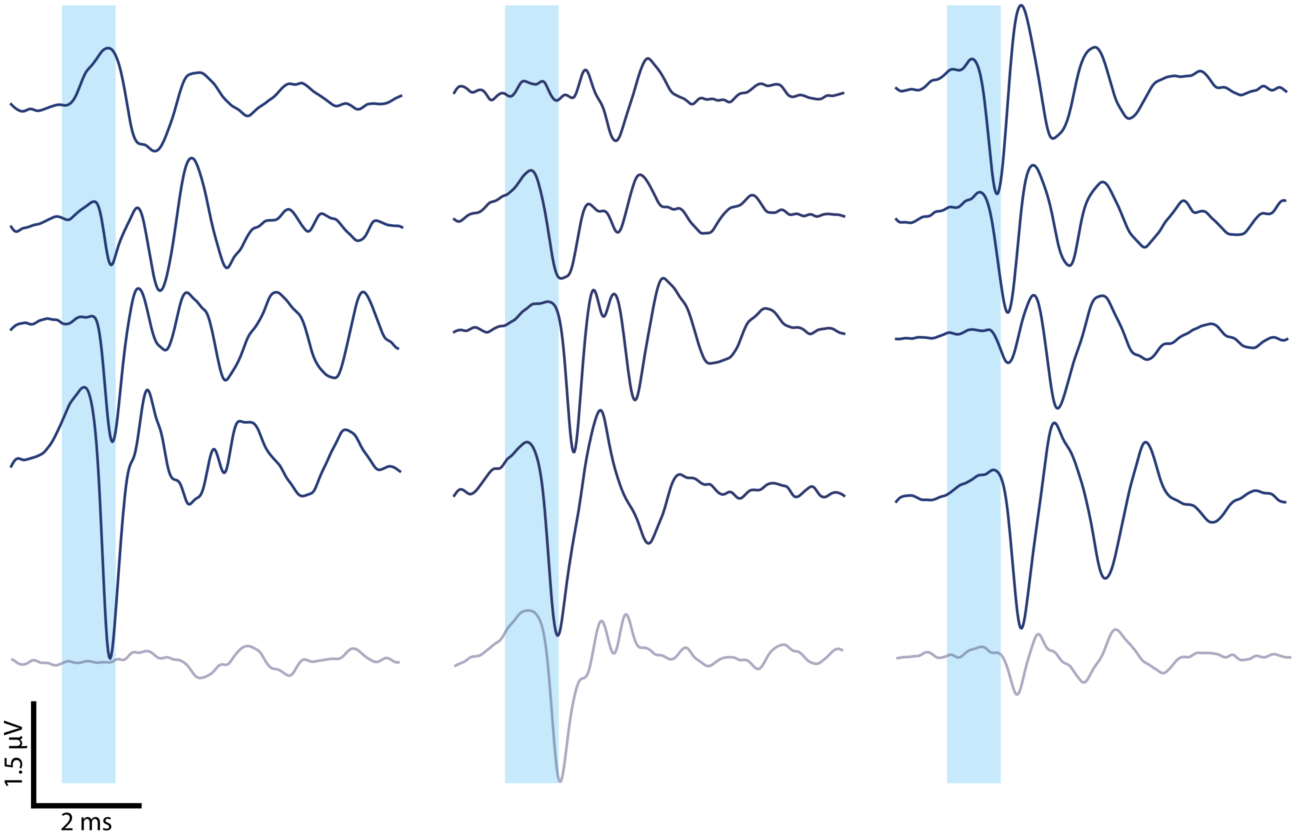

Figure EV1. Optically evoked auditory brainstem responses.

Functional opsin expression was verified in all *CatCh*-injected animals by recordings of optically evoked activity in the auditory nerve and brainstem driven by optical fibers at the start of each experiment before stimulating SGNs with oCIs for inferior colliculus recordings. Optical stimulation occurred with laser pulses of ~35 mW radiant flux and 1 ms pulse duration at a stimulation rate of 10 Hz delivered from a 200 µm optical fiber inserted via the round window. Each trace represents the average of 1,000 stimulus presentations in one animal. The oABR on the top right is also shown in Fig 1A. Gray oABRs were recorded from animals, from which no IC data could be obtained (due to death of the animal or misplacement of the electrode array).

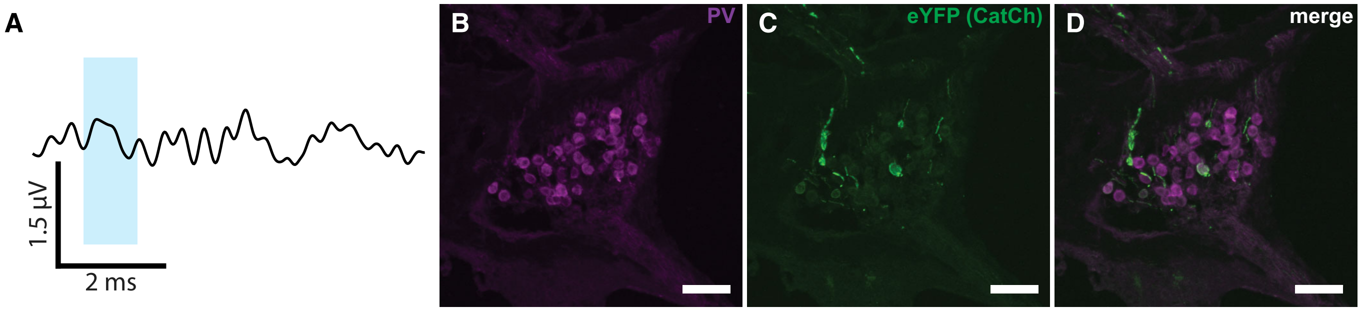

Figure EV2. oABR-negative gerbil.

A oABR (recording parameters as in Fig EV1) with no detectable response.
B–D *Catch*-eYFP-staining revealed transduction in only very few SGNs identified by parvalbumin expression (B) and *CatCh*-eYFP (C). Scale bar: 50 µm.

© 2020 The Authors

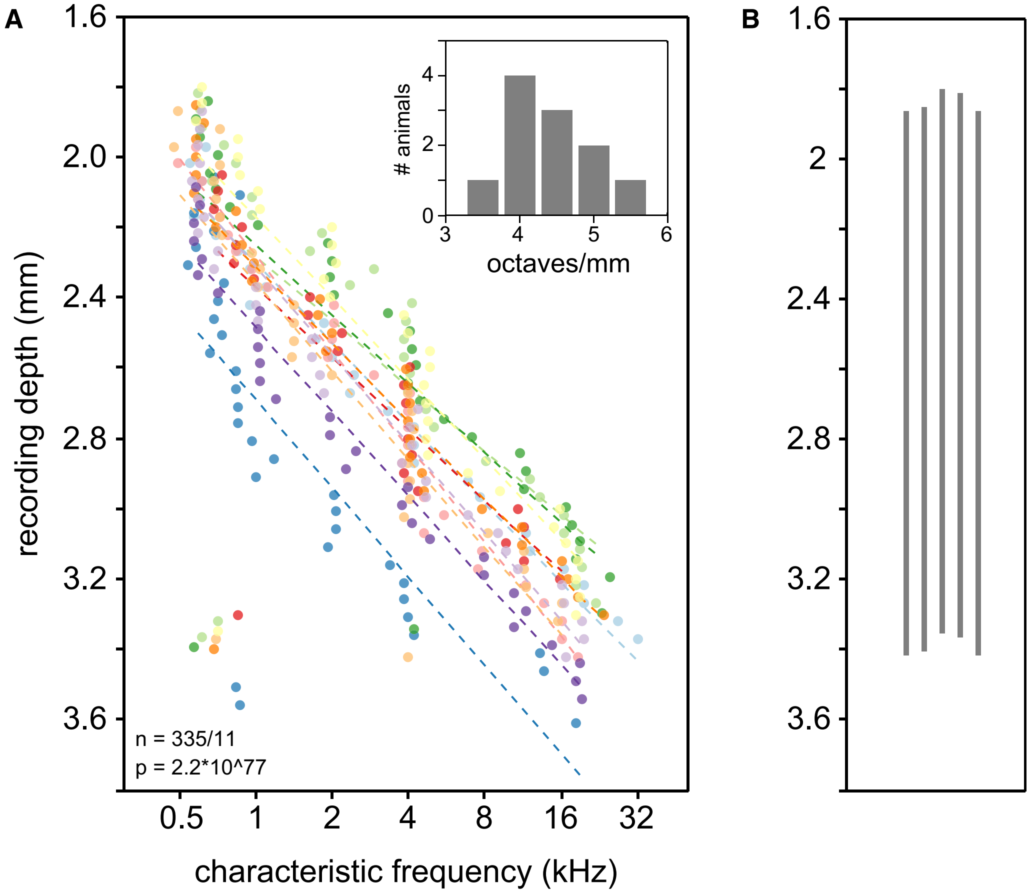

Figure EV3. Tonotopy in the auditory midbrain.

A Characteristic frequencies as a function of recording depth (normally distributed jitter of 0.05 octaves was added to each unit to reduce overlay of data points for better visualization). Tonotopic slopes (dashed lines) were calculated by linearly fitting characteristic frequencies at given recording depths for each animal (N = 11). Data from different animals are indicated by different colors. Inset: Distribution of tonotopic slopes of the 11 regular hearing animals contributing to this study.

B Electrode position in deafened animals (N = 5). The silicon probe was placed as in normal hearing animals, but tonotopy of the ICC could not be mapped.

© 2020 The Authors

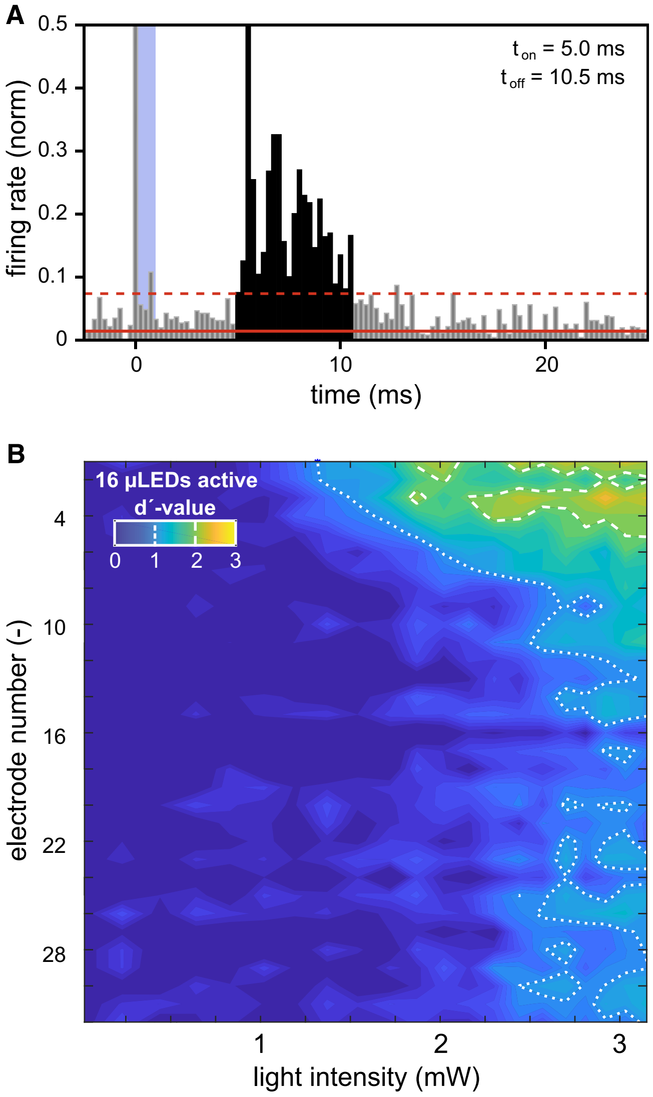

Figure EV4. oCI responses in non-injected gerbils.

A Peri-stimulus time histogram in response to SGN stimulation with 16 active µLEDs of an oCI at maximum radiant flux (~3.15 mW) in an exemplary recording with optically evoked ICC activity. Stimulus presentation is indicated in blue. Solid and dashed red lines indicate the mean firing rate plus 3 SD, respectively. Bin size was set to 0.25 ms, and the detected neural response is indicated in black. Responses have longer latencies when compared to oCI stimulation in virally transduced animals (compare to Appendix Fig S2C).

B Exemplar STCs in response to SGN illumination with all µLEDs in a non-injected gerbil. Responses are much weaker when compared to optogenetically manipulated animals and tonotopically do not correspond to the pattern of SGN illumination, as responses occur mainly in the dorsal, low-frequency regions of the ICC, even though basal, high-frequency regions of the cochlea were stimulated (compare to Fig 2A–C and G). Furthermore, these responses were not stable over time and could not be reproduced, as they vanished after the first recordings.

© 2020 The Authors
