# Supplementary material for: μLED‐based optical cochlear implants for spectrally selective activation of the auditory nerve
Source: EMBO Mol Med. 2020 Jun 29;12(8):e12387. doi: 10.15252/emmm.202012387 (PMC7411546; doi:10.15252/emmm.202012387)
Supplement: Supplementary file 3 — Code EV1 [file EMMM-12-e12387-s003.zip › MATLABcodes_analysis_final/readme_final.DOCX]

Each analysis file takes data from a folder corresponding to the file. This is hard coded, so if you work on a repository, download data to your own drive, or similar, you will have to change the name of the path in each filename (path\file). Some scripts contain multiple datasets (e.g. hearing vs deaf, wildtype vs CatCh-injected, …). The condition to be analyzed can be chosen by (un)commenting the desired datasets.

The name of each script, which might produce a datafile, figure, or both, starts with “RUN…”. M-files that are not named like this are functions which are called within the “RUN” script.

1. 00_calibration: “RUN_uLED-calibration outputs you the values and figures for µLED illumination when individual µLEDs are driven or all µLEDs are driven together. Blockwise µLED illumination is estimated based on the calibration curve of individual µLEDs.
2. 01_tonotopy:
   1. “RUNanalysis_tonotopy_hearing” outputs values and figures of the tonotopic slopes and their distribution recorded in each animal without pharmacological deafening. It also saves these slopes, which will later be used to estimate the spread of excitation upon optogenetic stimulation.
   2. “RUNanalysis_plot_recordingdepth_deaf” plots the recording depth in deafened animals. As no acoustic stimulation was possible here, there is no output saved. This script is simply producing a figure.
3. 02_PSTH_uLEDs: “RUN_PSTH_all_LED” produces PSTHs and calculates the time window of significant responses. It was used to determine the response window of all CatCh-transduced animals (hearing and deaf) in response to the strongest illumination using all µLEDs at once. The same was done for the wildtype animals which showed responses.
4. 03_a-d_STC_...: “RUNanalysis_STC_...”: These scripts perform the d’ analysis for stimulation using an optical fiber, individual µLEDs, blocks of µLEDs or all µLEDs. Essentially these scripts do the same thing, however the underlying data structure mildly differs between fiber stimulation and µLED stimulation. The reason for multiple µLED-scripts (dependent on the amount of active µLEDs) is that blockwise and individual µLED stimulation loops through the 4/16 different patterns of stimulation. In each script, different datasets can be chosen by (un)commenting the variable “files2analyze”) in the beginning of the script. Correspondingly, the line “save” with the corresponding filename to be saved needs to be uncommented at the end of each script, in order to save the analysis of different datasets with different filenames. The saved files will later be called in different scripts.
5. 04_plot_uLED_response_characteristics : “RUN_plot_dprime_analysis” plots the results of the scripts 03a-d. Besides visualizing these results, the script also performs statistical analysis to compare response characteristics between hearing and deafened animals (t-tests), thresholds of fiber- vs µLED-stimulation (ranksum), and the increase in response strengths and number of active electrodes for different stimulation modes (anova and multiple comparisons). These values can be displayed in the command window.
6. 05_tonotopic_activation: “RUN_tonotopy_block “and “RUN_tonotopy_single” analyze the tonotopic activation (i.e. best electrode as a function of emitter location), and calculate potential correlations. Both scripts contain some hard coded variables which describe the implant pitch of the oCIs used in the corresponding datasets, which should not be changed. In addition, RUN_tonotopy_single contains a variable that defines the oCI layout (i.e. arrangements of µLEDs) which should not be changed. Both scripts save the results in a .mat-file. The script “RUN_plot_tonotopy” takes these .mat-files and creates a figure that includes the data of both datasets.
7. 06_spread_of_excitation: “RUN_analyze_spectral_spread” analyzes the spectral spread, i.e. the space covered by responding electrodes, at desired d’ values at the best electrode. It also saves intermediate results for visual inspection (even though the figure quality is quite poor, and always scaled to the full range of stimuli, so one might have to zoom in). It saves the spatial spread sorted by µLED-pitch of the implants as well as the IDs of the animal that this data was recorded from, so it can be converted into spectral spread by using the tonotopic slopes calculated earlier. The scripts “RUN_plot_spectral_spread” and “RUN_plot_spectral_spread_octaves” read in this data (and in the _octaves script as well the tonotopic slope data, which has to be moved to this folder), plot the data, and perform an ANOVA with posthoc multiple comparison test to check for differences between implants of different µLED-pitch.

The final code has been tested in MATLAB R2019b and must potentially be adapted for use in other versions.
